# On contemporary aspects of assisted suicide at plato

**DOI:** 10.1192/j.eurpsy.2021.1195

**Published:** 2021-08-13

**Authors:** A. Voinov

**Affiliations:** Faculty Of Political Sciences, Philosophy And Communication Sciences, West University of Timisoara, Timisoara, Romania

**Keywords:** Plato, philosophy, Assisted Suicide

## Abstract

**Introduction:**

Usually, Plato is not considered a philosopher that comprehensively treated the matter of suicide. By studying Plato’s work (especially Crito, Phaedo, the Republic and the Laws), we observe that Plato was concerned with the problem of suicide and that he gave an elaborate answer regarding the problem of suicide, laws against its practice as well as exceptions from them, customs and punishments.

**Objectives:**

This paper, in the light of a trial to overcome the monistic approaches of the matter of suicide, proposes the modest but fundamental goal to point out the resemblance between Plato’s position (especially from the Laws and the Republic) regarding the matter of suicide and the nowadays reasons invoked by the patients requesting assisted suicide.

**Methods:**

Looking at the patients from the United States of America which requested assisted suicide, by analyzing the available annual reports (at the time of writing this abstract, only 6 out of 9 states that have a legal status that permits assisted suicide are publishing annual reports regarding the patients and their assisted suicide requests), we compare them with Plato’s attitude towards suicide.

**Results:**

We observe that the most invoked reasons (concerns and underlying illnesses), by the patients wich request assisted suicide, are also the cases in which Plato permitted suicide.

**Conclusions:**

This comparison and insight into Plato’s philosophy does not resolve any particular issues of the medical praxis but is binging out the utility of a multidisciplinary, especially philosophical and ethical, approach to the practice of assisted suicide.

